# Reablement in community-dwelling older adults: a randomised controlled trial

**DOI:** 10.1186/s12877-015-0142-9

**Published:** 2015-11-04

**Authors:** Hanne Tuntland, Mona Kristin Aaslund, Birgitte Espehaug, Oddvar Førland, Ingvild Kjeken

**Affiliations:** Department of Global Public Health and Primary Care, University of Bergen, Faculty of Medicine and Dentistry, P.O. Box 7804, 5018 Bergen, Norway; Bergen University College, Centre for Care Research Western Norway, P.O. Box 7030, 5020 Bergen, Norway; Bergen University College, Centre for Evidence-based Practice, P.O. Box 7030, 5020 Bergen, Norway; Haraldsplass Institute of Nursing, Deaconess University College, Ulriksdal 10, 5009 Bergen, Norway; Diakonhjemmet Hospital, National Advisory Unit on Rehabilitation in Rheumatology, P.O. Box 23, Vinderen, 0319 Oslo, Norway; Oslo and Akershus University College of Applied Sciences, Program of Occupational Therapy, Prosthetics and Orthotics, P.O. Box 4, St. Olavs plass, 0130 Oslo, Norway

**Keywords:** Rehabilitation, Activities of daily living, Older adults, COPM, Randomised controlled trial

## Abstract

**Background:**

There has been an increasing interest in reablement in Norway recently and many municipalities have implemented this form of rehabilitation despite a lack of robust evidence of its effectiveness. The aim of this study was to investigate the effectiveness of reablement in home-dwelling older adults compared with usual care in relation to daily activities, physical functioning, and health-related quality of life.

**Methods:**

This is a parallel-group randomised controlled trial conducted in a rural municipality in Norway. Sixty-one home-dwelling older adults with functional decline were randomised to an intervention group (*n* = 31) or a control group (*n* = 30). The intervention group received ten weeks of multicomponent home-based rehabilitation. The Canadian Occupational Performance Measure (COPM) was used to measure self-perceived activity performance and satisfaction with performance. In addition, physical capacity and health-related quality of life were measured. The participants were assessed at baseline and at 3- and 9-month follow-ups.

**Results:**

There were significant improvements in mean scores favouring reablement in COPM performance at 3 months with a score of 1.5 points (*p* = 0.02), at 9 months 1.4 points (*p* = 0.03) and overall treatment 1.5 points (*p* = 0.01), and for COPM satisfaction at 9 months 1.4 points (*p* = 0.03) and overall treatment 1.2 points (*p* = 0.04). No significant group differences were found concerning COPM satisfaction at 3 months, physical capacity or health-related quality of life.

**Conclusion:**

A 10-week reablement program resulted in better activity performance and satisfaction with performance on a long-term basis, but not the other outcomes measured.

**Trial registration:**

The trial was registered in ClinicalTrials.gov November 20, 2012, identifier NCT02043262.

**Electronic supplementary material:**

The online version of this article (doi:10.1186/s12877-015-0142-9) contains supplementary material, which is available to authorized users.

## Background

The growth in the ageing population, in combination with an expected shortage of health-care personnel in developed countries, present a huge challenge to the containment of future health-care costs [1]. A radical rethink of health-care services is required in order to address this challenge. As a consequence, there has been an increasing interest in home-care re-ablement services (hereafter ‘reablement’) in recent years [2, 3]. The term ‘reablement’ is used in the UK [4], Ireland [2], and Denmark [5], whereas this form of rehabilitation is known as ‘restorative care’ in the US [6], Australia [7], and New Zealand [8]. The two terms are however, regarded as synonyms [2, 9–11]. Reablement is a timely approach to improve home-care services for older people needing care or experiencing functional decline. The health-care providers are organised into an integrated, coordinated multidisciplinary team whose members work together with the person towards shared goals [12]. The intervention is targeted, multicomponent and intensive, and takes place in the person’s home and local surroundings. The focus is on enhancing performance of daily activities defined as important by the person. The aim is to increase independence in daily activities, and enable people to age in place, be active and participate socially and in the society.

The effects of reablement on Personal Activities of Daily Living (PADL) have been summarised in a systematic review [13], in which five trials were included. The authors concluded that there is some, but limited, evidence that reablement can reduce the home-care service users’ dependency in PADL. Further, the effects of reablement have been evaluated in three randomised controlled trials (RCTs). In an Australian RCT with a 12-month follow-up, reablement was compared with usual care [11]. The trial reported outcomes such as PADL, Instrumental Activities of Daily Living (IADL), physical functioning, risk of falls and health-related quality of life. The results showed no differences between groups in individual outcomes over time, except for improvement in IADL at the 12-month follow-up for the reablement group. In addition, two RCTs were conducted in New Zealand. The first RCT investigated social support and physical functioning and found improved physical functioning in favour of reablement [8]. The second RCT investigated health-related quality of life and demonstrated significant differences in favour of reablement [14]. In summary, the research on the effectiveness of reablement is scarce and the results are inconsistent.

To the best of our knowledge, 28 % of Norwegian municipalities have implemented reablement during the last 3 years despite a lack of robust evidence of its effectiveness. In this first RCT on reablement conducted in Europe, our aim was to evaluate whether reablement is more effective with regard to self-perceived activity performance and satisfaction with performance, physical functioning, and health-related quality of life compared with usual care.

## Methods

### Study design and setting

We performed a parallel-group randomised controlled superiority trial in which all participants were assessed at baseline, and after 3 and 9 months. We conducted the study in a primary care setting in a rural municipality in Norway with approximately 14,000 inhabitants. The recruitment period lasted from May 2012 until February 2014. The intervention group was offered reablement and the control group was offered usual care. The study complies with the CONSORT statement [15] for transparent reporting (see Additional file 1) and is registered November 20, 2012 in ClinicalTrials.gov, identifier NCT02043262. The study protocol has been published previously [16].

Ethics approval for the study was granted by the Regional Committees for Medical and Health Research Ethics in Norway (REK West, 2012/295). All participants received information about the study and gave written consent. The research was carried out in accordance with the Declaration of Helsinki.

### Participants

People applying for, or referred to, home-based services were potential participants for the study based on their self-reported activity limitations. Some of the participants had been hospitalised due to an acute illness, while others were recruited after having gradually developed functional decline not needing hospitalisation or institution-based treatment. We included home-dwelling persons over the age of 18 years, who lived in the municipality, were able to understand Norwegian, and had a functional decline in one or more daily activities. We excluded people if they were in need of institution-based rehabilitation or a nursing home placement, were terminally ill, or were moderately or severely cognitively reduced (subjectively assessed by health-care providers based on observation and communication.

### Randomisation and blinding

The randomisation with an allocation ratio of 1:1 using a computer-generated permuted block randomisation sequence, with randomly selected block sizes of lengths 2 and 4, was performed by a biostatistician not involved in the assignment of participants to groups. We concealed the allocation sequence in sequentially numbered, opaque, sealed envelopes. The allocation list was stored in a safe deposit box in a central office in the municipality. Neither health-care providers enrolling participants nor research assistants had influence on group allocation. The research assistants conducted the baseline assessments in the participant’s home prior to randomisation. The participants were urged not to reveal their group allocation to the research assistants during follow-up assessments. The success of the research assistants’ blinding was recorded. Researchers conducting data entry and data analysis were blinded to group allocation.

### Interventions

#### Reablement

The Canadian Model of Occupational Performance and Engagement (CMOP-E) [17] matches the client-centred reablement intervention and was used as a theoretical framework in the study. In CMOP-E, occupational performance is perceived as the result of interaction and interdependence between the person(s), the environment, and the occupation(s). Accordingly, the primary outcome was measured by the Canadian Occupational Therapy Performance (COPM), which was developed as part of the first version of the CMOP-E [17]. COPM is a client-centred tool to enable individuals to identify and prioritise everyday issues that restrict or impact their performance in everyday living. COPM focuses on enabling people to perform activities they experience as difficult, but important in their daily life. As a consequence, the therapeutic process is tailored according to the needs and aims of the individual participant, resulting in differences in the number and type of elements in the intervention across participants, as described elsewhere [16]. However, the intervention consisted of both general and individual features. Among the general features was a maximum rehabilitation period of 3 months. Further, as part of baseline assessments, the occupational therapist and physical therapist used the COPM to identify activity limitations perceived as important by the participant. Thereafter, this information was used to develop a rehabilitation plan. The therapists supervised the home-care personnel, some of whom had no formal education (assistants), in how to encourage and assist the person in the daily training. The focus was on stimulating the participants to perform the daily activities themselves, rather than letting others do it for them. Among the individual features were training in daily activities, adaptations to the environment or the activity, and exercise programs.

All health-care personnel attained training before the intervention was implemented, in particular in the ideology of self-management. The therapists took courses and were instructed in how to conduct the assessments. The therapists had weekly informal lunch meetings with the home-care staff in order to ensure good communication and follow-up of individual participants. Simpler physical exercises or skills training the assistants could provide, were illustrated and described in a booklet in the participant’s home and also demonstrated during the informal meetings. New staff members were given extra attention in order to ensure adherence to the treatment.

#### The control intervention

Usual care was chosen as the comparator, as this is the conventional treatment offered to homebound persons in most municipalities in Norway. For most participants, usual care meant receiving the compensating help they applied for, in terms of personal or practical assistance, safety alarm, meals on wheels, or assistive technology. However, for a few participants, it comprised rehabilitation assisted by an occupational therapist (*n* = 1) and/or physical therapist (*n* = 5) based on the participants’ own efforts. Hence, the usual care was also diverse. Usual care was not time-limited, and persisted after the 3 months intervention period if needed.

### Outcome measures

Socio-demographic characteristics were collected at baseline. We used four different outcome measures, which were collected at the three measurement time points. Co-interventions were registered for hospital admissions, institution-based rehabilitation, day centre placement, and outpatient treatment at both follow-ups. Work hours allocated to home-based services and distribution of health-care professions were collected daily during the first 3 months. A detailed description of measurements and outcomes collected are published in the protocol [16].

#### Primary outcome

Self-perceived activity performance and satisfaction with that performance were measured by the COPM [17]. During a semi-structured interview, the participant was encouraged to identify problems with his/her self-care, productivity and leisure activities. The participant rated the importance of each identified activity (range 1 to 10, 10 = extremely important). Thereafter, the participant prioritised and rated the five most important activities in performance and satisfaction with performance again on 1 to 10-point scales (higher scores reflect better performance or higher satisfaction). For the reablement group, the rehabilitation goals were the prioritised activities, hereafter termed ‘activity goals’. The activity goals identified by the control group were only used for evaluation purposes. We calculated two mean sum scores based on the performance and satisfaction scores of the activity goals in COPM, respectively. According to the COPM manual, a difference of 2 points in the mean sum score is regarded as either a clinically relevant improvement or deterioration [17].

#### Secondary outcomes

We measured functional mobility using the Timed Up and Go test, which is an observer-based instrument originally developed as a short test of basic mobility skills in frail community-dwelling elderly persons [18]. Normative values for community-dwelling older adults with 1.8 medical diagnoses aged 70–79 years is 9 s for both men and women [19]. The cut-off value for independent transfer in community-dwelling older adults with a variety of medical conditions is < 20 s [18].

We measured grip strength in kilograms with the hydraulic instrument, Jamar Dynamometer, according to a standard protocol [20]. Normative grip strength in a healthy community-dwelling population aged 70–79 years, is 42.4 k and 23.7 k for men and women respectively, for the right hand, and 40.5 k and 22.0 k, respectively, for the left hand [21].

Health-related quality of life was measured by the COOP/Wonka, which is a generic, self-reported outcome measure [22]. We chose the revised version [23], which consists of six questions with associated drawings, where each question represents a separate domain. The responses were scored on a five-point ordinal scale ranging from 1 to 5 (1 = best, 5 = worst).

### Statistical analysis

The calculation of sample size was based on the results from an earlier study performed on older adults, in which the standard deviation for the primary outcome was 1.4 for COPM performance and 1.6 for COPM satisfaction [24]. With an assumed standard deviation of 2.5 and a within-subject correlation coefficient of 0.7, we estimated that 42 participants were needed to detect a change of 2 points as statistically significant (with a two-sided 5 % level and a power of 80 %). As a high dropout rate of up to 40 % could be expected due to the potential frailty of the participants, we decided to include 60 participants (30 people in each group).

All participants were analysed according to initial group allocation (intention-to-treat). Differences at baseline between participants in the two groups were analysed by the independent samples t-test for means, the χ^2^ test for proportions, and exact test when assumptions were not met. These tests were also applied in the co-intervention analysis and in the analysis of usage of home-based services and distribution of health-care professions. Treatment effects (mean differences between the groups at 3 months and 9 months, and for the overall effect for the total trial period) were estimated with mixed-effects models [25], with adjustments for baseline measurements. Group and time by group interaction were entered as fixed factors, time as a repeated factor and participant as a random factor. Models were fitted with random intercepts and with random intercepts in combination with random slopes for time. Likelihood-ratio tests were performed to investigate whether a random slope improved model fit. If not, the simpler model was selected. Effect sizes defined as standardised mean differences (Cohen’s d) were computed at each time point. A simple adjustment for potential baseline group differences was performed by subtracting baseline effect sizes from effect sizes at follow-up. The analyses were performed using IBM SPSS Statistics version 22 and R [26]. *P*-values < 0.05 were considered statistically significant.

## Results

### Participants

**Fig. 1:**
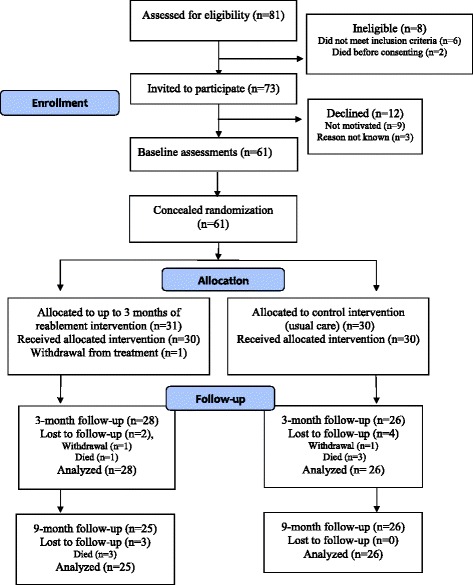
Flowchart of participants throughout the trial

Sixty-one participants were randomised to reablement (*n* = 31) or to usual care (*n* = 30). Due to continuous monitoring of missing data during the trial period, there were few missing outcomes data. The dropout rate was 11 % and 16 % at the 3-month and 9-months follow-ups respectively, and was mainly due to deaths among participants. The flow diagram of the study is outlined in Fig. 1. No adverse events related to treatment occurred during the data collection period.

Participants were primarily older females (69 %), who lived alone (77 %) and without higher education (84 %). The baseline Timed Up and Go, Jamar dynamometer and the COOP/Wonka physical fitness scores, together with the high number of deaths, indicate a frail sample with lower physical function than normative scores for community-dwelling persons aged 70–79 years. The total number of prescribed medications was equally distributed between the two groups at all measurement time points and stable during the 9-month follow-up period, with 6 and 7 medications in the reablement group and control group, respectively. Table 1 presents the baseline demographic characteristics by study group. Overall, the baseline characteristics were well matched between the groups.

**Table 1:** Baseline characteristics

Characteristics	Intervention (*n* = 31)	Control (*n* = 30)	*p*-value
Age, mean (SD), range	79.9 (10.4), 45	78.1 (9.8), 42	0.49
Female, no (%)	22 (71.0)	19 (63.3)	0.53
Married/cohabitating, no (%)	10 (32.3)	4 (13.3)	0.08
Education < university/university college, no (%)	27 (87.1)	24 (80.0)	0.51
Retired, no (%)	28 (90.3)	26 (86.7)	0.65
Motivation for rehabilitation, scale 1–10, 10 is best, mean (SD)	7.5 (2.3)	7.7 (2.1)	0.70
Total number of prescribed medications, mean (SD), range	6.1 (2.8), 13	6.7 (3.1), 11	0.46
Self-reported number of medical conditions, mean (SD), range	3.0 (1.7), 8	2.9 (1.1), 4	0.79
Category of main medical condition			0.42
Cardiovascular condition, no (%)	5 (16.1)	2 (6.7)	
Neurological condition included strokes, no (%)	8 (25.8)	8 (26.7)	
Orthopedic condition, no (%)	10 (32.3)	12 (40.0)	
Lung condition, no (%)	4 (12.9)	1 (3.3)	
Other/unspecified condition, no (%)	4 (12.9)	7 (23.3)	
Activity performance (COPM), sum score, scale 1–10, 10 is best, mean (SD)	2.6 (1.5)	2.8 (1.4)	0.70
Activity satisfaction (COPM), sum score, scale 1–10, 10 is best, mean (SD)	2.6 (1.6)	3.3 (1.9)	0.12
Mobility and balance (Timed Up and Go), seconds, mean (SD), (*n* = 56)	24.6 (11.9)	23.3 (17.3)	0.73
Grip strength (Jamar dynamometer), men right hand, kilograms, mean (SD), (*n* = 19)	24.4 (14.1)	28.8 (9.6)	0.43
Grip strength (Jamar dynamometer), men left hand, kilograms, mean (SD), (*n* = 17)	27.3 (13.4)	25.8 (9.0)	0.79
Grip strength (Jamar dynamometer), women, right hand, kilograms, mean (SD), (*n* = 39)	17.7 (5.7)	15.8 (6.6)	0.34
Grip strength (Jamar dynamometer), women, left hand, kilograms, mean (SD), (*n* = 41)	17.1 (6.7)	14.4 (6.1)	0.18
Physical fitness (COOP/Wonka ), scale 1–5, 1 is best, mean (SD)	4.4 (0.6)	4.2 (0.7)	0.29
Feelings (COOP/Wonka), scale 1–5, 1 is best, mean (SD)	2.4 (1.5)	2.3 (0.9)	0.71
Daily activities (COOP/Wonka), scale 1–5, 1 is best, mean (SD)	3.5 (1.1)	3.2 (0.8)	0.16
Social activities (COOP/Wonka), scale 1–5, 1 is best, mean (SD)	2.4 (1.4)	2.9 (1.3)	0.13
Change in health (COOP/Wonka), scale 1–5, 1 is best, mean (SD)	2.4 (1.0)	2.1 (0.9)	0.34
Overall health (COOP/Wonka), scale 1–5, 1 is best, mean (SD)	3.0 (0.9)	2.9 (0.8)	0.46

*SD* Standard deviation

N is only specified if less than 61 participants

Differences between groups were tested by using independent samples t-tests for means and χ^2^ for proportions (exact test when assumptions were not met)

**Fig. 2:**
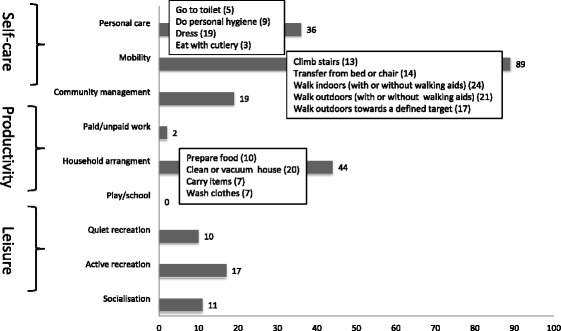
Number of prioritised (dark grey staples) activity limitations in 61 participants assessed with the Canadian Occupational Performance Measure (COPM). Activities described by >20 participants are listed under each category, with the number of participants who prioritised this activity in parentheses

In baseline COPM interviews, the participants described 297 activity limitations of which 228 were prioritised. The distribution of activity goals among the nine activity categories are illustrated in Fig. 2. The most frequent activity goal was to improve mobility.

### Intervention

Table 2 presents time registration data with a description of work hours allocated to home-based services and distribution of health-care professions during the first 3 months. For the reablement group the rehabilitation period lasted, on average, 10 weeks. There were no significant differences in the amount of home-based service work hours (*p*-values not shown). There were however significant differences in the distribution of health professionals (*p*-value <0.001). The higher emphasis on rehabilitation in the reablement group is reflected in the substantially higher number of home visits from therapists in this group, and also in the more diverse team composition in this group compared to the control group.

**Table 2:** Usage of home-based services and distribution of health-care professions during the first three months

Home visits and time usage	Intervention (*n* = 29)	Control (*n* = 23)
Mean home visits pr. person (no, SD)	78 (65)	71 (82)
Mean home visits pr. person pr. week (no, SD) ^a^	7 (5)	6 (7)
Mean hours home-based service pr. person (no, SD)^b^	24.7 (21.7)	20.1 (39.0)
Mean hours home-based service pr. person pr. week (no, SD)^a b^	2.1 (1.8)	1.7 (3.2)
Distribution of home visits among health-care professions	Intervention (*n* = 29)	Control (*n* = 23)
Nurse (%)	15.0	24.2
Auxiliary nurse (%)	35.0	43.2
Assistant (%)	22.7	24.0
Physical therapist (%)	9.9	2.6
Occupational therapist (%)	13.3	0.2
Social educator (%)	1.1	1.5
Speech therapist (%)	0.0	0.0
Student (%)	3.0	3.1
Unknown profession (%)	0.0	1.2
Mean number of professions involved pr. person^c^	5	3

*SD* Standard deviation

^a^Based on a 12-week data collection period

^b^Travel time excluded

^c^Students are excluded from analysis

We found a significantly higher number of co-interventions at the 3-month follow-up in the control group; 12 outpatient treatments in the control group versus 3 outpatient treatments in the intervention group (*p* = 0.007), of which 10 of the outpatient treatments were physiotherapy (data not shown).

### Primary outcomes

After 3 months, there was a significant mean difference in favour of the reablement group in the COPM performance score of 1.5 points (95 % CI: 0.3-2.8, *p* = 0.02) (Table 3). The difference was still significant at the 9-month follow-up with a mean difference of 1.4 points (95 % CI: 0.2-2.7, *p* = 0.03). Further, there was a significant overall treatment effect in the 9-month trial period of 1.5 points (95 % CI: 0.4-2.6, *p* = 0.01). While there were no significant differences between the groups in the COPM satisfaction scores after 3 months, the mean difference score at 9 months was 1.4 points (95 % CI: 0.4-2.7, *p* = 0.03), and the overall treatment score was 1.2 points (95 % CI: 0.1-2.3, *p* = 0.04) in favour of the reablement group. The effect sizes were moderate to large (range 0.7-0.9).

**Table 3:** Treatment effect of reablement versus usual care estimated with mixed-effects models^a^

	Reablement group	Control group	Adjusted effect size	Treatment effect, mean difference (95 % CI)	*p*-value	Overall treatment effect^d^, mean difference (95 % CI)	*p*-value
	Mean (95 % CI)	Mean (95 % CI)					
Activity performance ^e^ (COPM) (1–10, 10 is best performance)						1.5 (0.4-2.6)	0.01
Baseline	2.6 (2.1-3.2)	2.8 (2.2-3.3)	-	-	-	-	-
3 months^b^	6.9 (6.1-7.8)	5.5 (4.7-6.3)	0.8	1.5 (0.3-2.8)	0.02	-	-
9 months^c^	6.3 (5.0-7.6)	4.8 (4.1-5.5)	0.7	1.4 (0.2-2.7)	0.03	-	-
Activity satisfaction ^e^ (COPM) (1–10, 10 is best satisfaction)						1.2 (0.1-2.3)	0.04
Baseline	2.6 (2.0-3.2)	3.3 (2.6-4.0)	-	-	-	-	-
3 months^b^	6.7 (5.9-7.6)	6.0 (5.3-6.8)	0.7	1.0 (−0.3-2.2)	0.13	-	-
9 months^c^	6.5 (5.2-7.8)	5.2 (4.5-5.9)	0.9	1.4 (0.1-2.7)	0.03	-	-
Mobility and balance ^f^ (Timed up and Go) (seconds)						−0.1 (−3.8-3.5)	0.96
Baseline	24.6 (20.1-29.2)	23.3 (16.4-30.1)	-	-	-	-	-
3 months^b^	19.6 (14.2-25.1)	17.9 (14.0-21.8)	0.1	−0.4 (−4.3-3.5)	0.82	-	-
9 months^c^	19.9 (14.7-25.0)	18.1 (13.4-22.8)	0.1	0.3 (−3.7-4.3)	0.88	-	
Grip strength^e^ (Jamar dynamometer), right hand (kilograms)						−0.4 (−2.4-1.5)	0.66
Baseline	19.6 (16.2-23.0)	20.6 (16.6-24.5)	-	-	-	-	-
3 months^b^	20.1 (17.3-22.9)	20.6 (16.4-24.7)	0.1	−0.3 (−2.5-2.0)	0.81	-	-
9 months^c^	18.6 (15.4-21.8)	19.5 (15.3-23.7)	0.1	−0.6 (−2.9-1.7)	0.59	-	-
Grip strength ^e^ (Jamar dynamometer), left hand (kilograms)						−1.1 (−3.5-1.3)	0.36
Baseline	19.8 (16.1-23.5)	18.0 (14.6-21.5)	-	-	-	-	-
3 months^b^	20.8 (16.9-24.7)	20.0 (16.7-23.2)	−0.1	−0.1 (−3.1-2.8)	0.92	-	-
9 months^c^	19.4 (15.9-23.0)	20.4 (15.1-25.6)	−0.3	−2.2 (−5.2-0.9)	0.16	-	-
Physical fitness^fg^ (COOP/Wonka) (1–5, 1 is best)						−0.2 (−0.6-0.2)	0.34
Baseline	4.4 (4.2-4.7)	4.2 (4.0-4.5)	-	-	-	-	-
3 months^b^	4.0 (3.6-4.3)	3.9 (3.5-4.3)	−0.2	0.0 (−0.4-0.5)	0.94	-	-
9 months^c^	3.8 (3.4-4.2)	4.1 (3.8-4.5)	−0.6	−0.4 (−0.9-0.1)	0.09	-	-
Feelings^f^ (COOP/Wonka) (1–5, 1 is best)						0.0 (−0.5-0.5)	0.90
Baseline	2.4 (1.9-3.0)	2.3 (2.0-2.6)	-	-	-	-	-
3 months^b^	2.3 (1.8-2.7)	2.2 (1.7-2.7)	0.0	0.0 (−0.5-0.6)	0.89	-	-
9 months^c^	2.2 (1.7-2.6)	2.1 (1.7-2.5)	−0.1	0.0 (−0.6-0.6)	1.00	-	-
Daily activities^f^ (COOP/Wonka) (1–5, 1 is best)						−0.4 (−0.8-0.1)	0.14
Baseline	3.5 (3.1-3.9)	3.2 (2.9-3.5)	-	-	-	-	-
3 months^b^	2.7 (2.3-3.1)	2.9 (2.5-3.2)	−0.6	−0.4 (−0.9-0.2)	0.21	-	-
9 months^c^	2.5 (2.0-3.0)	2.8 (2.3-3.2)	−0.6	−0.4 (−0.3-0.5)	0.22	-	-
Social activities^f^ (COOP/Wonka) (1–5, 1 is best)						0.3 (−0.3-0.8)	0.35
Baseline	2.4 (1.9-2.9)	2.9 (2.4-3.4)	-	-	-	-	-
3 months^b^	2.3 (2.0-2.7)	2.2 (1.7-2.6)	0.6	0.4 (−0.2-1.0)	0.23	-	-
9 months^c^	2.3 (1.7-2.8)	2.3 (1.9-2.7)	0.4	0.1 (−0.5-0.8)	0.65	-	-
Change in health^f^ (COOP/Wonka) (1–5, 1 is best)						0.0 (−0.3-0.3)	0.78
Baseline	2.4 (2.0-2.7)	2.1 (1.8-2.5)	-	-	-	-	-
3 months^b^	2.8 (2.5-3.1)	2.6 (2.4-2.9)	0.0	0.1 (−0.2-0.5)	0.40	-	-
9 months^c^	3.0 (2.9-3.2)	3.1 (2.9-3.4)	−0.4	−0.1 (−0.4-0.3)	0.66	-	-
Overall health^f^ (COOP/Wonka) (1–5, 1 is best)						−0.2 (−0.6-0.2)	0.31
Baseline	3.0 (2.7-3.4)	2.9 (2.6-3.2)	-	-	-	-	-
3 months^b^	2.8 (2.5-3.0)	2.9 (2.5-3.2)	−0.3	−0.2 (−0.6-0.2)	0.36	-	-
9 months^c^	2.8 (2.4-3.1)	2.9 (2.6-3.3)	−0.4	−0.2 (−0.6-0.2)	0.40	-	-

*CI* Confidence interval

^a^Adjusted for baseline values

^b^Treatment effect is the estimated mean group difference at the 3-month follow-up

^c^Treatment effect is the estimated mean group difference at the 9-month follow-up

^d^ Overall treatment effect is the estimated mean group difference for the whole trial period of 9 months

^e^ Positive values favour the reablement group

^f^ Negative values favour the reablement group

### Secondary outcomes

There were no significant differences between the groups in any of the secondary outcomes after 3 or 9 months, nor in the overall mean difference scores. However, both groups improved in their mobility/balance and in most of the health-related quality of life domains, and these effects were sustained at the 9-month follow-up (Table 3). Grip strength did not improve in either of the groups.

Blinding of research assistants had a success rate of 63 % at the 3-month and 64 % at the 9-month follow-up.

## Discussion

The main aim of this study was to evaluate whether reablement is more effective than usual care with regard to self-perceived activity performance and satisfaction with activity performance, physical functioning, and health-related quality of life. The results demonstrate that home-dwelling older adults with functional decline benefit from reablement in terms of improving their self-perceived performance and satisfaction with performance in prioritised daily activities. Furthermore, these health effects were sustained on a long-term basis.

The COPM treatment effects for COPM performance of 1.4 -1.5 points are both below the cut-off value of 2 points (a 22 % change), being a clinically relevant change reported in the COPM manual [17]. However, evidence to support this cut-off value is lacking. The significant difference between groups of 1.4 points in the current study does, however, equal the optimum threshold for improvements for performance scores reported in a previous study of responsiveness of the COPM [27], and has also been used as an estimate of a clinically relevant difference in another trial [28]. Nonetheless, more studies are needed in order to establish the clinically relevant change of COPM.

As shown in Table 2, the weekly intensity of the reablement intervention was quite low. However, an important finding in this study was that despite the fact that no extra time resources were allocated to the reablement group, significant improvements were found in COPM performance and satisfaction with performance compared to the control group. This is contrary to the expectation that implementation of reablement requires more resources than usual care during the rehabilitation phase [29]. However, even though the total time resources were similar between groups, the reablement group had more therapy time and less nursing time compared to the usual care group.

Interestingly, the control group also reported increased levels of activity performance and satisfaction with performance. The same phenomenon has been reported in previous studies, where the authors suggest that the improvement may be caused by the therapeutic effect of the baseline COPM interview, which increases the control group’s awareness of their activity limitations and prompts them to seek solutions themselves [28, 30]. Another explanation is the phenomenon of spontaneous recovery after an episode of functional decline. Many of the participants had fractures, where a spontaneous recovery after surgery is expected. Hence, a subgroup analysis of this group would have been interesting in order to explore this issue further. However, the sample was too small for such analysis. The improvements in the control group may also have been caused by contamination from the intervention arm of the study to the control arm. Due to problems with recruitment in a sparsely inhabited municipality, the intervention was implemented in all home-care districts in the municipality. Thus, it was not possible to avoid the situation where the same health-care personnel provided both the experimental and control interventions, however to different participants. Also, the significantly higher amount of co-interventions in terms of outpatient physiotherapy received by participants in the control group during the first 3 months might have had an impact.

Despite the significant improvements in activity performance and satisfaction with performance, there were no differences between the two groups in functional mobility, grip strength, or health-related quality of life over the trial period. This is in contrast to another trial with a similar intervention who found improvement in physical function after reablement [8]. However, our study was only statistically powered to find results for the primary outcome. As a result, the small sample size with the control group improving as well, does not rule out a Type 2 error. However, it is well established that there is a complex relationship between body functions and activity performance [31], in which physical performance such as muscle strength correlates only moderately with activity performance [32]. Reablement is directed at achieving personal activity goals. Thus, an important intervention component is to perform the specified activities in the participant’s home environment with health-care professionals present. The positive effect on activity performance in the reablement group may therefore be caused by factors such as increased confidence in performing activities, and by optimising performance through adaptations of the activity and/or the environment.

The study was conducted in a real-life context in primary care. Even if the inclusion criteria permitted participation of persons over the age of 18 years, the sample turned out to be an aged, heterogeneous population with comorbidities and a wide range of functional decline. Hence, the results may not be generalisable to an adult population with other characteristics.

A strength of this study is that we used a patient-specific outcome measure to ensure congruence between participants’ needs, therapy priorities, intervention and evaluation. Further, COPM allowed each participant to choose and rate the activity limitations he/she considered important. As a consequence, the ‘noise’ that frequently occurs in standardised instruments related to fixed items experienced as irrelevant by participants was reduced, thereby increasing the responsiveness for capturing the effects of reablement. Additional strengths are that researchers performing data entry and data analysis were blinded. Although outcomes were collected on a long-term basis, few outcomes data were missing and the dropout rate at the 9-month follow-up was low. Further, all outcomes in the study are reported according to the protocol.

Methodological limitations of this study are similar to those of many other rehabilitation trials in that participant and health-care provider blinding was impossible. The blinding of research assistants at follow-ups was not completely successful. Further, all co-interventions were not equally distributed between the groups. Treatment fidelity, i.e. if the treatment was delivered as intended [33], was not adequately monitored. Consequently, we do not know whether assistants delivered the intervention as intended. Moreover, the compliance to the interventions was not systematically recorded, and there was a possibility of contamination from one arm of the study to the other.

## Conclusions

In this study, reablement was found to be a superior intervention to usual care in terms of improving self-perceived activity performance and satisfaction with performance on a long-term basis in community-dwelling older adults. However, the other outcomes measured showed no significant group differences. The intervention was given to a frail, elderly population, who still demonstrated a significant improvement despite no extra time resources being allocated.

## Additional file

Additional file 1:
**Consort checklist.** (DOC 218 kb)

## Abbreviations

CONSORT: Consolidated standards of reporting trials
COPM: Canadian occupational performance measure
IADL: Instrumental activities of daily living
PADL: Personal activities of daily living
RCT: Randomised controlled trial
